# Analytical validation of protein biomarkers for risk of spontaneous preterm birth

**DOI:** 10.1016/j.clinms.2017.06.002

**Published:** 2017-06-12

**Authors:** Chad Bradford, Rob Severinsen, Trina Pugmire, Matison Rasmussen, Kathryn Stoddard, Yuta Uemura, Spencer Wheelwright, Marija Mentinova, Daniel Chelsky, Stephen W. Hunsucker, Paul Kearney, Durlin Hickok, Tracey C. Fleischer, Ilia Ichetovkin, J. Jay Boniface, Gregory C. Critchfield, John M. Peltier

**Affiliations:** a Sera Prognostics, Inc., 2749 East Parleys Way, Suite 200, Salt Lake City, UT 84109, USA; b Caprion Biosciences, 201 Avenue Président Kennedy, Suite 3900, Montréal, Québec H2X 3Y7, Canada; c Integrated Diagnostics, Inc., 219 Terry Avenue North, Suite 110, Seattle, WA 98101, USA

**Keywords:** Spontaneous preterm birth, Proteomics, Mass spectrometry, Insulin-like growth factor-binding protein 4, Sex hormone-binding globulin, Validation, Clinical

## Abstract

Presented are the validation results of a second-generation assay for determining the relative abundances of two protein biomarkers found in maternal serum that predict an individual’s risk of spontaneous preterm birth. The sample preparation workflow is complex, consisting of immuno-depletion of high-abundance serum proteins, tryptic digestion of the immuno-depleted fraction to generate surrogate peptide analytes, and detection by tandem mass spectrometry. The method was determined to be robust on observation of the following characteristics: classifier peptide detection precision was excellent; results were accurate when compared to a reference method; results were linear over a clinically relevant range; the limits of quantitation encompassed the range of expected results; and the method demonstrated analytical specificity and resilience to differences in patient serum and common endogenous interferents.

## Highlights

• Results for an assay validation detecting two spontaneous preterm birth biomarkers.
• Sample prep of immuno-depletion, tryptic digest, and mass spectrometric detection.
• Precision, accuracy, linearity, LOQ and analytical specificity determined.
• Method provides robust means of determining relative abundances of biomarkers.
• Allows for prediction of individual risk of spontaneous preterm birth.

## 1 Introduction

Preterm birth (PTB), defined as delivery at fewer than 37 weeks of gestation, is the leading cause of mortality and morbidity in neonates [1]. Worldwide, PTB impacts 15 million deliveries annually and results in over one million infant deaths [2], [3]. Spontaneous onset of preterm birth (sPTB) represents a high percentage of all PTB cases [4]. Until recently, sPTB lacked an adequate prognostic test. The development of such a test was complicated by the variety of etiologic associations described for sPTB, including infection, inflammation, placental complications, and uterine distension [4]. The complex etiology requires that an effective prognostic test must have the ability to interrogate multiple biological pathways.

Increasingly, proteomics is being used in clinical diagnostic testing as a predictors for a variety of complex diseases and conditions (e.g., spontaneous preterm birth [5], lung cancer [6], therapeutic targeting of breast cancer [7])*.* These conditions are often characterized by numerous and diverse interconnecting biological pathways, requiring systematic approaches for the development of comprehensive clinical diagnostic tests. Tandem mass spectrometry is not only capable of multiplexing assays, but it can also be rigorously validated on a wide variety of analytes, including proteins [8], [9], [10], [11], [12], [13].

We applied a targeted proteomics workflow, coupled with highly multiplexed tandem mass spectrometry detection, to simultaneously monitor peptides from candidate signature proteins and quality control proteins in subsets of clinical study serum samples. Insulin-like growth factor-binding protein 4 (IBP4) and sex hormone-binding globulin (SHBG) were previously shown by our group to perform well as biomarkers for discriminating sPTB from term birth [5]. Additionally, we utilized this technology to develop and clinically validate a bivariate protein biomarker assay for the qualitative prediction of individual risk of spontaneous preterm birth [5].

We previously validated the first-generation assay using an Agilent 6490 and a 30-min liquid chromatography gradient. This method established acceptable measures of analytical validation, including precision, carryover, limit of detection, and analytical specificity, and was used to assay blinded samples during a previously published clinical validation [5] and commercial samples later. As with any new assay, performance requirements change over time, either to improve analytical performance or reduce cost. To increase the laboratory's throughput and to enable integration of a current-generation mass spectrometer, a plan was developed to validate the method after migration to an Agilent 6495 triple quadrupole mass spectrometer with a 15-min liquid chromatography gradient. The validation plan required that this novel second-generation assay demonstrate analytical performance equivalent to that of the previously validated [5] first-generation method, prior to being placed into clinical practice. Validation of the second-generation clinical diagnostic assay for the IBP4 and SHBG signature proteins, which measured precision, alternative method comparison, linearity, limits of quantitation, carryover, analytical specificity, interference, and stability [14], is described herein.

## 2 Experimental

### 2.1 Materials

#### 2.1.1 Validation samples and quality control material

This work used samples derived from the Proteomic Assessment of Preterm Risk (PAPR) study, which enrolled 5501 pregnant women from 11 clinical sites and is broadly representative of the US population [15]. Maternal serum was collected from patients between 17 and 28 weeks of gestation for the purposes of developing a second trimester serum-based test predictive of sPTB risk. Women with singleton pregnancies, aged 18 to 60 years, receiving prenatal care and capable of providing consent, were eligible for the study. Subjects pregnant with more than one fetus or those with a known or suspected fetal anomaly were excluded. Of the 5235 women who continued the study, pre-specified exclusions resulted in the removal of 326 subjects with medically-indicated preterm birth, 109 subjects who had been treated with progesterone, and 28 samples with pre-analytical issues (e.g., hemolysis). Of the remaining samples, 413 were used for analytical validation. The PAPR study followed the Good Clinical Practice guidelines issued by the International Conference on Harmonisation [16].

In addition to the PAPR samples, two pools of serum from female donors were purchased from Golden West Biologicals (Temecula, CA). One serum pool (QC1) was created from equal volumes of serum from 10 non-pregnant female donors. A second serum pool (QC2) was created from equal volumes of serum from 21 pregnant donors. The QC pools were a critical part of quality control for each batch, allowing for long-term trend analysis of assay performance. Non-pooled serum from individual donors was also purchased from Golden West Biologicals. Both the pooled materials and the single donor samples consisted of a large number of identical single-use aliquots that were stored in the same conditions as clinical samples (−80 °C). The pooled materials and single-donor samples were collected using the same protocol used in the PAPR study.

Phosphate buffered saline served as a negative control sample and was purchased from Life Technologies (Carlsbad, CA).

#### 2.1.2 Reagents and consumables

Protein Depletion Buffer A and B were supplied by Agilent Technologies (Santa Clara, CA). Acetonitrile and water were LC-MS grade and were purchased from ThermoFisher (Hampton, NH). Methanol was HPLC grade and was purchased from JT Baker. Dithiothreitol (DTT), iodoacetamide (IAA) and trifluoroacetic acid (TFA) were purchased from Sigma (St. Louis, MO). A custom order of solubilized Trypsin Gold was purchased from Promega (Madison, WI). Formic acid was purchased from ThermoFisher (Hampton, NH).

High purity stable isotope standards (SIS) were purchased from New England Peptides (Gardner, MA). The SIS were checked for a minimum purity of 95% by HPLC. A mass spectral analysis was also performed and yielded an acceptable measured mass within 0.1% of the calculated average molecular weight. An amino acid analysis was performed to verify the amino acid composition to within 20% of the theoretical concentration for each amino acid and to determine the molar yield of each peptide. Carboxy-terminal lysine and arginine residues of SIS peptides were uniformly labeled with ^13^C or ^15^N resulting in either a +8 or +10 amu mass shift, respectively. Individually synthesized peptides were used to create a pool of high purity SIS containing heavy-labeled analogues of signature and quality control peptides monitored in the PreTRM® assay. SIS peptides were used at a final concentration that approximated the abundance of the endogenous peptides, excepting the linearity and limits of quantitation studies.

### 2.2 Methods

#### 2.2.1 Initial workflow and assay development

Early assay development involved the creation of an MRM assay to perform relative determination of abundances of 242 proteins chosen because of their association in the literature with preterm birth and other pregnancy complications. Public and proprietary databases were used to identify up to five proteotypic peptides per protein based on previous detection in blood. The assay was further refined by supplementation with novel discoveries, recurrent literature curation, and trimming of the assay size using peptide correlation and analytical performance. Mass spectrometer settings were optimized for each peptide to provide the highest signal-to-noise, the highest precision, or a signal free of chromatographic anomalies. Work flow optimization considered both serum and plasma blood fractions, protein depletion strategies and materials, tryptic digestion conditions, solid phase extraction-based desalting methods, LC-MS/MS gradient separation, and HPLC column performance. Initial assay development utilized separate injections of synthetic unlabeled peptides to confirm analyte identity. Later phases of assay development utilized sample fortification with high purity heavy-labeled peptide standards to confirm analytical specificity.

#### 2.2.2 Batch design

Except for the batch runs to determine linearity and limits of quantitation, a standardized plate batch design was adopted and used for this work. The batch design utilized replicates of the two serum quality control pools (i.e., QC1 and QC2). Two terminal replicates of phosphate buffered saline, named Process Blanks, served to monitor for routine carryover and cross contamination. A maximum of 24 clinical samples were assayed in a single batch, as shown in Table 1, representing a standardized batch design; run order was top to bottom, column 1 to column 4.

**Table 1:** Standard batch design.

	Column 01	Column 02	Column 03	Column 04
Row A	QC1 Replicate 1	Clinical Sample	Clinical Sample	Clinical Sample
Row B	QC2 Replicate 1	Clinical Sample	Clinical Sample	Clinical Sample
Row C	Clinical Sample	Clinical Sample	Clinical Sample	Clinical Sample
Row D	Clinical Sample	Clinical Sample	Clinical Sample	Clinical Sample
Row E	Clinical Sample	Clinical Sample	Clinical Sample	QC1 Replicate 3
Row F	Clinical Sample	Clinical Sample	Clinical Sample	QC2 Replicate 3
Row G	Clinical Sample	QC1 Replicate 2	Clinical Sample	Process Blank 1
Row H	Clinical Sample	QC2 Replicate 2	Clinical Sample	Process Blank 2

#### 2.2.3 Protein depletion chromatography

Clinical serum samples and pooled serum quality control materials were retrieved from −80 °C storage and allowed to thaw while resting on crushed wet ice. Once thawed and mixed by inverting three times, 50 µL of a serum sample was added to a well of a 0.2 µm polypropylene filter plate (Captiva Filter Plate, Agilent Technologies, Santa Clara, CA) containing 150 µL of Protein Depletion Buffer A. The plate was mixed on a plate vortexer for approximately 30 s and then vacuum filtered into a polypropylene deep 96-well plate.

The sample filtrates were depleted of 14 high abundance proteins using a protein depletion system based on Agilent 1260 liquid chromatography instrumentation running ChemStation software (B.04.03 SP2). Sample filtrates in the deep well plate were maintained at 4 °C in an autosampler. A 100 µL injection of the sample filtrate was fractionated on a protein depletion column (Agilent MARS14, 4.6 × 100 mm) at 23 °C and a flow rate of 0.125 mL/min of Protein Depletion Buffer A. The flow-through fraction containing signature proteins was detected with a peak at 11.0 min by UV absorption at 280 nm and was collected in a polypropylene deep 96-well plate controlled at 4 °C (fraction collection time was 8.00–14.00 min, approximate volume of fraction was 0.8 mL). After the flow-through fraction was collected, the bound fraction was eluted to waste by pumping Protein Depletion Buffer B through the depletion column at 1 mL/min. Once the bound fraction was eluted from the column, as confirmed by detection of UV absorption at 20.5 min, the column was re-equilibrated with Protein Depletion Buffer A at 1 mL/min for 11 min. The flow rate was reduced to 0.125 mL/min of Protein Depletion Buffer A to condition the depletion column for the next cycle. The cycle time was 36 min.

**Fig. 1:**
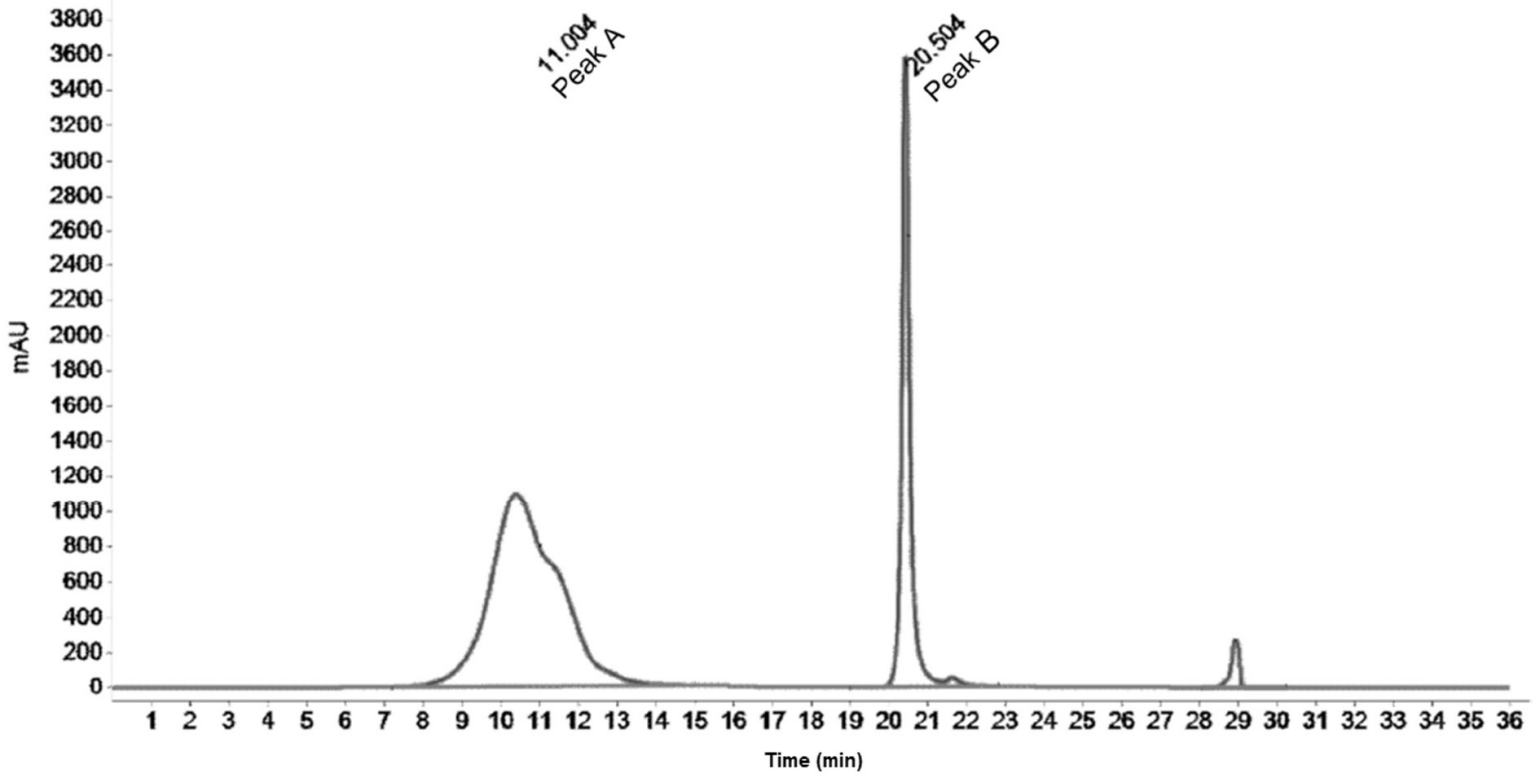
Representative depletion chromatogram.

Protein depletion was monitored by calculating the ratio of the chromatographic peak area of the proteins of interest (Peak A) to the chromatographic peak area of the depleted proteins (Peak B). A representative chromatogram is shown in Fig. 1.

#### 2.2.4 Enzymatic digest

Serum samples that were depleted of high abundance proteins underwent a trypsin digestion to generate proteotypic peptides that served as surrogate analytes for the proteins. The depleted samples were fortified with acetonitrile to a final concentration of 5%. Dithiothreitol (DTT), from a single-use aliquot stored at −20 °C, was added to each sample to a concentration of 5 mM and mixed by pipetting; the samples were then incubated at 60 °C for 20 min in a water bath. After the DTT incubation, the sample plate was equilibrated to room temperature (the lab temperature is set to 23 °C year-round). Iodoacetamide (IAA), from a single-use aliquot stored at −20 °C, was added to a concentration of 5 mM, mixed by pipetting, and incubated at room temperature for 30 min in the dark. After the IAA incubation, trypsin was added to a concentration of 6.25 µg/mL and mixed by pipetting (approximate enzyme:substrate of 1:40). The samples were then incubated at 34.5 °C for 17 h. Digested samples were fortified with a pool of high purity stable isotope standards (SIS) [17], [18]. The samples were mixed and then split into two equal 0.345 mL fractions. One fraction continued through the remainder of the work flow and the second was held at −80 °C in reserve for repeat analysis, as needed.

#### 2.2.5 Solid phase extraction-based desalting

Samples were acidified by adding TFA to a final concentration of 1.5% (v/v), followed by solid phase extraction to remove buffer salts. A 96-well solid phase extraction plate (Empore, 3 M, St. Paul, MN) fitted to a vacuum manifold was conditioned with 0.1 mL of methanol, then 0.2 mL of water, then 0.3 mL of 5% acetonitrile in water (v/v). The entire volume of the sample was added to the conditioned plate. The sample was washed with 0.3 mL of 5% acetonitrile in water (v/v). Peptides were eluted with 0.25 mL of 95% acetonitrile in water (v/v). The eluate was frozen at -80 °C and then lyophilized to dryness.

#### 2.2.6 LC-MS/MS analysis

Lyophilized desalted samples were reconstituted in 25 µL of a 2% acetonitrile, 0.1% formic acid in water solution (v/v), which matched the initial LC gradient conditions. An injection volume of 10 µL of sample was separated on an Agilent 1290 UPLC system fitted with a Poroshell EC-C18 (2.1 × 50 mm, 2.7 µm) reversed-phase LC column maintained at 50 °C. The mobile phases used in the separation were: (A) 0.1% formic acid in water (v/v), and (B) 0.1% formic acid in acetonitrile (v/v). The flow rate was maintained at 0.4 mL/min. The LC gradient used the following linear steps (time, %B): 0.00 min, 2%; 0.25 min, 2%; 4.00 min, 9%; 12.50 min, 35%; 13.00 min, 95%; 14.00 min, 95%; 14.50 min, 2%; 15.00 min, end. There was a 2.50 min equilibration at initial conditions prior to the next injection. The LC eluent was diverted to waste for the initial 1.8 min and after 13.4 min post-injection to minimize MS source fouling. The cycle time between sample injections was 17.50 min. The reconstituted samples were maintained at 4 °C in the autosampler while awaiting analysis.

Peptides were monitored on an Agilent 6495 triple quadrupole mass spectrometer operating with a Jet Stream electrospray ionization source in positive ion mode (MassHunter B.08.00). Unit resolution was used for both the first and second mass filtering quadrupoles. Source parameters were: gas temperature at 150 °C, gas flow at 11 L/min, nebulizer pressure at 20 psi, sheath gas temperature at 350 °C, sheath gas flow at 11 L/min, capillary at 4000 V, high pressure funnel RF at 200 V, and low pressure funnel RF at 110  V.

Data was acquired with a dynamic MRM method using a 1 min retention time window for the signature analytes and 0.5 min window for all other peptides. Each peptide was monitored with two correlating transitions. In addition to the two signature peptides (Table 2), the method monitored a panel of quality control peptides, as well as a large number of peptides that are of research and development interest and not required for calculating sPTB risk. The total number of transitions monitored was 442 with the method yielding average dwell times for the IBP4 and SHBG signature peptides of 42 and 50 ms, respectively, and a maximum number of concurrent transitions of 60 (Appendix A).

**Table 2:** PreTRM signature analyte LC-MS/MS settings.

Protein	Proteotypic Peptide	Analyte Type	Transition	Retention Time (min)	Collision Energy	Dwell Time (ms)	Peptide Transition	SIS Transition
IBP4	QCHPALDGQR	Signature	Quantitative	2.64	11	42	394.5_475.2	397.9_485.2
IBP4	QCHPALDGQR	Signature	Qualitative	2.64	17	42	394.5_360.2	397.9_370.2
SHBG	IALGGLLFPASNLR	Signature	Quantitative	10.82	13	50	481.3_657.4	484.6_667.4
SHBG	IALGGLLFPASNLR	Signature	Qualitative	10.82	17	50	481.3_412.3	484.6_412.3

**Fig. 2:**
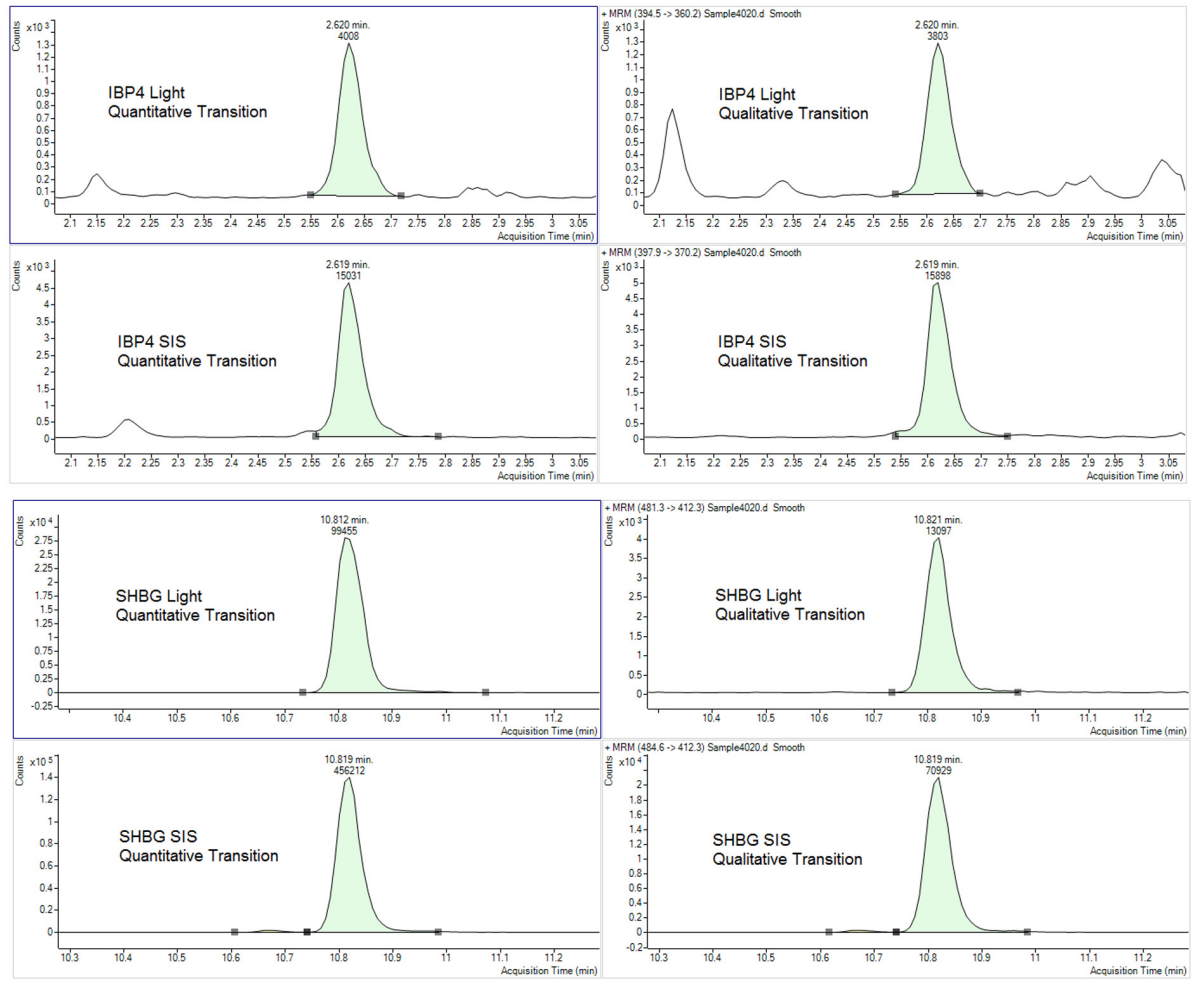
Peptide chromatography.

Chromatographic peak areas of the quantitative transition were determined using MassHunter Quantitative Analysis software (Agilent, B.07.01). Representative chromatograms are shown in Fig. 2.

#### 2.2.7 Risk of spontaneous preterm birth calculation

The laboratory LIMS system (Nautilus LIMS (v 9.2), ThermoFisher) calculated the response ratio for the peptides according to Eqs. (1) and 2 by dividing the chromatographic area counts (*a*_1_ and *a*_2_) of the signature peptide by the chromatographic area counts of the corresponding SIS (*b*_1_ and *b*_2_) for IBP4 and SHBG, respectively.

(1) $$R_{1}=\frac{a_{1}}{b_{1}}$$

(2) $$R_{2}=\frac{a_{2}}{b_{2}}$$

The LIMS subsequently determined the ratio of these peptide response ratios, which corresponded to the relative change in serum levels of the two marker proteins. The proteomic score, *S*, is the natural logarithm of the ratio of relative response ratios of IBP4 (numerator) to SHBG (denominator):

(3) $$S=\ln\left(\frac{R_{1}}{R_{2}}\right)$$

The risk of delivery before 37 weeks of gestation is reported as a Bayesian posterior probability based on the patient’s individual proteomic score, *S* [5]. The algorithm utilizes the relative abundances of the signature proteins and the patient’s body mass index (BMI) to generate a qualitative risk score [5].

#### 2.2.8 Precision

The precision of the assay was assessed using individual runs of the same sample sets on multiple instrumentation clusters, multiple lots of reagents, and multiple operators. Intra-batch precision (i.e., repeatability) and inter-batch precision (i.e., reproducibility) were determined by running a sample set (n = 6) repeatedly in twenty-one randomly ordered batches over a span of 34 days. The sample set was comprised of the samples shown in Table 3 with four replicates of each sample being run in each batch.

**Table 3:** Precision samples.

Sample identification	Sample characteristic
15–6036	High IBP4 Peptide, High SHBG Peptide
QC1	Low SHBG Peptide
15–6018	Low IBP4 Peptide
15–6054, 15–6092, 15–6138	Midrange Proteomic Score

Precision acceptability was determined using the analyte response ratio %CV. Establishing appropriate acceptance criteria was accomplished by modeling changes in the response ratio of signature analytes and determining if those changes had a clinically relevant impact on the calculated risk of sPTB.

#### 2.2.9 Alternative method comparison

Results of accuracy relative to clinical outcome have previously been published [5] using data that was generated by the first-generation validated method (data not shown). The analytical accuracy of the second-generation PreTRM assay (with Agilent 6495 detection and reduced LC run time) was assessed using an alternative method comparison to the first-generation method (with Agilent 6490 detection and a longer LC run time). The alternative method comparison involved assaying 413 individual PAPR samples in 21 batches with each batch split into two equivalent fractions after trypsin digestion (Section 2.2.4). One set of fractions was analyzed on two Agilent 6490 s running the longer LC method. The other set was analyzed on three Agilent 6495 s running the shorter LC method. Agilent 6495 derived signature analyte response ratios and proteomic scores versus the same data derived from the Agilent 6490 s were compared using linear regression.

#### 2.2.10 Linearity and limits of quantitation

To complete the linearity and limits of quantitation experiments, a sufficient number of replicates of the healthy pregnant donor serum pool were processed through the trypsin digestion step and then pooled. The pool was fortified with high concentrations of SIS peptides and then serially diluted with additional volumes of the pooled, partially-processed serum sample. The replicates, n = 10 for each concentration level, were then continued through the remainder of the work flow and submitted for LC-MS/MS analysis. The resulting samples maintained a constant concentration of endogenous analytes and a wide range of concentrations of SIS. A reversed response ratio was calculated by dividing the chromatographic peak areas of the SIS response by the chromatographic peak areas of the endogenous peptide. Linearity and limits of quantitation were determined on three Agilent 6495 LC-MS/MS systems.

#### 2.2.11 Analytical specificity

The ability of the assay to consistently measure the two signature analytes in maternal serum was assessed using transition ratios. The mass spectrometric method had two mass transitions for each analyte. If a serum sample contained an endogenous or exogenous substance that interfered with the detection of a transition, the ratio of the responses from the two transitions was abnormal. The transition ratio was calculated using Eq. (4).

(4) Transition Ratio=(Chromatographic Peak Area of Qualitative Transition/Chromatographic Peak Area of Quantitative Transition)∗100

#### 2.2.12 Endogenous interferent testing

The potential for common endogenous interferents to impact the ability of the assay to measure either of the signature peptides was evaluated by fortifying an intended use sample (QC2) with high concentrations of triglyceride-rich lipoprotein, hemolysate, protein (i.e., albumin and immunoglobulins), conjugated bilirubin, or unconjugated bilirubin. A commercially sourced endogenous interferent kit (Assurance Interference Test Kit, Sun Diagnostics, New Gloucester, ME) was used to fortify QC2 to create the test pool. The test pool was compared to a control pool that was created using QC2 diluted with the solvent system used to prepare the interferent stock. Equal numbers of test and control pool replicates (n = 7) were assayed using the complete work flow, and the proteomic score was calculated for all replicates. A two-tailed homoscedastic *t*-test was used to determine if the p-value of the proteomic score differences between the test and control pools exceeded a predefined ≥0.05 acceptance criteria.

#### 2.2.13 Analyte stability

Individual serum samples (n = 12, Golden West Biologicals) were subjected to multiple freeze/thaw cycles to determine the impact on signature analytes as interpreted by the proteomic score. Serum samples from twelve healthy pregnant donors, received and stored at -80 °C, were subjected to three freeze/thaw cycles. A thaw cycle involved the retrieval of the sample from −80 °C storage and incubation at ambient temperature (i.e., 23 °C) for approximately one hour. The subsequent freeze cycle was for at least 21 h at −80 °C. A single analytical batch was used to assay the stressed sample and a non-stressed aliquot of the same sample. The proteomic score was calculated and a paired *t*-test was used to determine if the mean of the 12 stressed samples was significantly different (p value ≤ 0.05) than the mean of the 12 unstressed samples.

## 3 Results and discussion

### 3.1 Intra-Batch and Inter-Batch precision

**Fig. 3:**
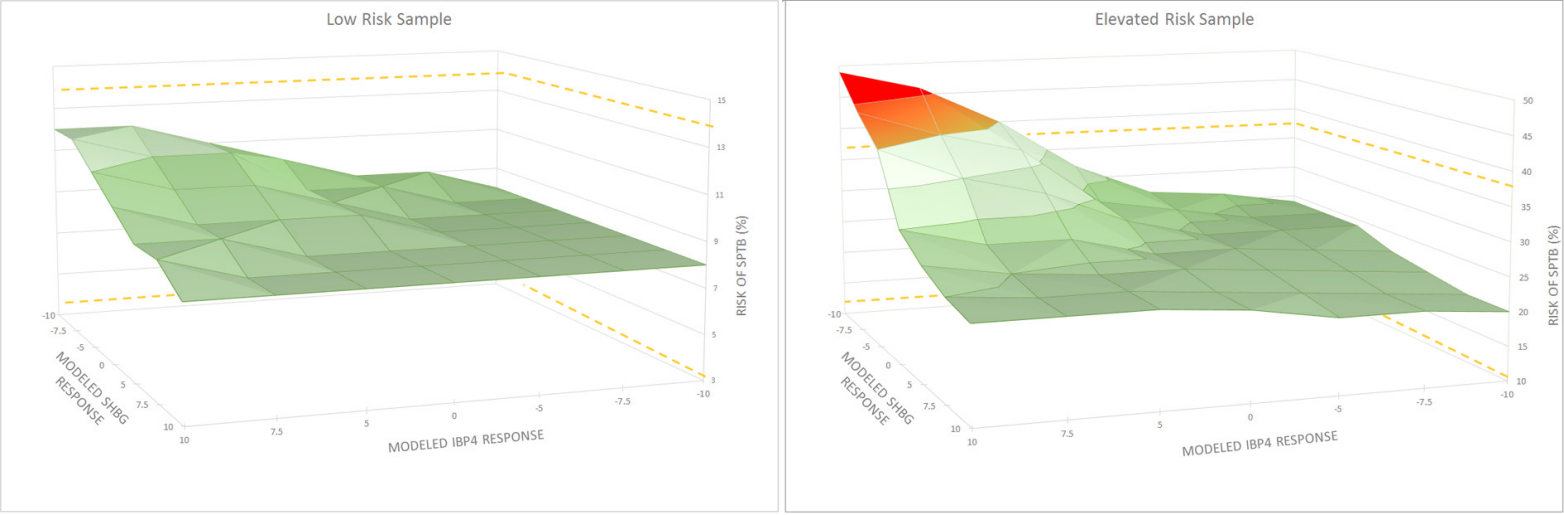
Modeling impact of analyte response variability on calculated risk of sPTB.

The assay was designed to utilize relative abundances of IBP4 and SHBG to assign a risk for sPTB. The lowest reported risk of sPTB is <7.3% which represents the prevalence rate of sPTB in the United States based on the data and methodology at the time of clinical validation [19]. The highest reported risk is >60% [5]. A confidence interval around risk of sPTB was generated during clinical validation [5]. To determine acceptable precision performance, two of the precision samples were used to model analyte response variance up to ±10%. One of the precision samples (15–6036, Table 3) was from a subject who had a risk of sPTB at the US prevalence rate (i.e., <7.3%) as previously determined multiple times (n = 9). The other precision sample (15–6138, Table 3) was from a subject who had a risk of sPTB that was over three times the US sPTB prevalence rate (i.e., 24%), also previously measured multiple times (n = 3). Surface plots for these two samples were generated with x- and y-axes representing modeled IBP4 and SHBG response ratios, respectively (Fig. 3). The sample mean analyte response ratio was set at zero on each axis (center of surface plots). The risk of sPTB, plotted on the z-axis, was calculated using combinations of modeled analyte response ratios and assessed as to whether the modeled values were outside of the risk confidence interval determined in clinical validation (dashed lines along the z-axis plane, [5]). For the sample at the US sPTB prevalence rate, all combinations of modeled analyte response ratios resulted in risks of sPTB that were within the established risk confidence interval (e.g., no risk of a false positive result for a lower risk sample). For the elevated risk sample, there was a change in the calculated risk that exceeded the risk confidence interval when the IBP4 response ratio was high in conjunction with a low SHBG response ratio, as indicated in the red portion of the plot. While such a situation cannot absolutely be ruled out, the most common analytical errors (e.g., digestion, sample or SIS handling) would change both signature analyte responses in the same direction and would be corrected by the relative protein response used to calculate the proteomic score. Nevertheless, the change in determined risk of sPTB did not affect the predictive value of the result; the elevated risk status was maintained (e.g., no risk of a false negative result for an elevated risk sample).

Based on the precision modeling results, a 20% CV for each signature analyte response ratio was deemed to not significantly affect the risk of sPTB determination. Therefore, 20% CV for analyte response ratio was used as the acceptance criteria for intra-batch precision and inter-batch precision.

After repeated analysis of six precision samples in 21 batches, four replicates of each sample per batch, the response ratio for each signature analyte was assessed for agreeable results. For intra-batch precision, the established acceptance criteria were that for each of the six precision samples tested, the signature analytes had response ratio %CVs within a batch that were ≤20% in 20 of the 21 batches (≥95%). Each of the precision samples had at least 20 of 21 batches with IBP4 and SHBG response ratio %CVs that were ≤20% (Table 4). Two data points were dropped from the calculations because of a sample handling error that resulted in the samples being combined into a single well. A third data point was dropped because of a trypsin digestion anomaly.

**Table 4:** Intra-batch precision results.

Sample	PRECISION_01		PRECISION_02		PRECISION_03		PRECISION_04		PRECISION_05		PRECISION_06		PRECISION_07	
	SHBG	IBP4	SHBG	IBP4	SHBG	IBP4	SHBG	IBP4	SHBG	IBP4	SHBG	IBP4	SHBG	IBP4
15–6018	4.63	7.89	5.10	5.12	6.62	11.97	6.41	1.90	10.40	7.76	5.20	13.01	3.02	6.43
15–6036	7.26	2.72	1.50	5.22	4.42	9.71	2.56	4.37	9.92	7.04	4.08	7.77	6.77	12.98
15–6054	1.97	5.47	9.20	7.96	8.38	6.79	4.65	9.38	8.54	4.84	4.95	9.84	2.29	9.98
15–6092	5.98	5.73	16.62	7.87	2.16	4.38	3.70	10.94	*21.42*	10.87	4.25	7.80	4.38	6.69
15–6138	3.83	9.82	8.79	7.96	8.45	6.71	4.96	4.03	9.85	7.59	5.27	3.61	3.19	6.79
Precision QC1	5.90	4.02	12.52	10.89	7.51	7.64	19.29	19.76	*22.16*	13.28	9.75	5.79	5.35	2.80

Sample	PRECISION_08		PRECISION_09		PRECISION_10		PRECISION_11		PRECISION_12		PRECISION_13		PRECISION_14	
	SHBG	IBP4	SHBG	IBP4	SHBG	IBP4	SHBG	IBP4	SHBG	IBP4	SHBG	IBP4	SHBG	IBP4
15–6018	7.04	7.07	8.91	11.09	8.46	5.62	3.35	8.67	9.54	15.52	7.43	8.67	7.71	5.66
15–6036	7.07	4.77	5.69	7.21	6.00	6.29	8.60	6.96	8.85	10.91	4.44	7.06	5.59	8.26
15–6054	8.01	13.61	4.93	8.19	1.63	2.25	6.31	3.47	16.82	14.34	14.37	3.19	7.26	5.30
15–6092	4.07	7.18	6.86	8.35	2.63	11.64	4.82	10.78	7.54	6.27	19.52	9.16	7.61	4.97
15–6138	6.60	10.46	7.20	5.18	2.80	1.77	5.42	5.82	9.90	2.35	3.90	3.37	1.44	3.77
Precision QC1	10.67	11.29	7.63	9.88	3.71	11.43	2.76	3.77	12.12	5.34	16.91	9.81	7.69	9.09

Sample	PRECISION_15		PRECISION_16		PRECISION_17		PRECISION_18		PRECISION_19		PRECISION_20		PRECISION_21	
	SHBG	IBP4	SHBG	IBP4	SHBG	IBP4	SHBG	IBP4	SHBG	IBP4	SHBG	IBP4	SHBG	IBP4
15–6018	4.55	8.90	10.53	7.64	1.16	3.42	4.66	10.60	6.32	7.63	11.15	12.88	7.51	4.87
15–6036	4.17	2.15	7.81	12.31	9.18	13.38	10.59	8.18	10.53	4.64	6.70	7.94	4.19	6.84
15–6054	8.66	6.92	3.32	9.85	*21.64*	11.35	14.90	5.29	3.64	6.81	5.57	4.53	1.89	8.57
15–6092	6.16	6.81	3.56	6.62	4.67	2.41	17.27	5.73	5.00	5.02	5.56	7.86	8.33	9.35
15–6138	10.96	15.13	2.37	2.67	0.98	11.12	14.30	6.38	3.07	10.55	11.96	9.81	5.71	3.53
Precision QC1	4.86	5.10	2.07	6.93	5.88	7.95	10.00	9.92	3.49	8.59	4.70	10.46	3.36	9.06

Italicized values were over the 20% CV acceptance criteria.

The same 21 batches were used to assess inter-batch precision. For inter-batch precision, the established acceptance criteria were that for each of the six precision samples, the signature analytes had response ratio %CVs across all 21 batches that were ≤20%. Each of the precision samples had IBP4 and SHBG response ratio %CVs that were ≤20% (Table 5).

**Table 5:** Inter-batch precision results.

Sample	Sample Characteristics	N	SHBG%CV	IBP4%CV	Mean SHBG	Mean IBP4
*Reproducibility*						
15–6018	Low IBP4	84	9.67	11.41	1.103	0.192
15–6036	High IBP4, High SHBG	83	9.73	9.49	1.766	0.323
15–6054	Midrange Proteomic Score	84	11.76	10.05	0.861	0.264
15–6092	Midrange Proteomic Score	83	11.89	9.52	0.882	0.285
15–6138	Midrange Proteomic Score	83	9.00	8.87	0.909	0.310
Precision QC1	Low SHBG	84	12.71	10.22	0.188	0.253

### 3.2 Alternative method comparison

**Fig. 4:**
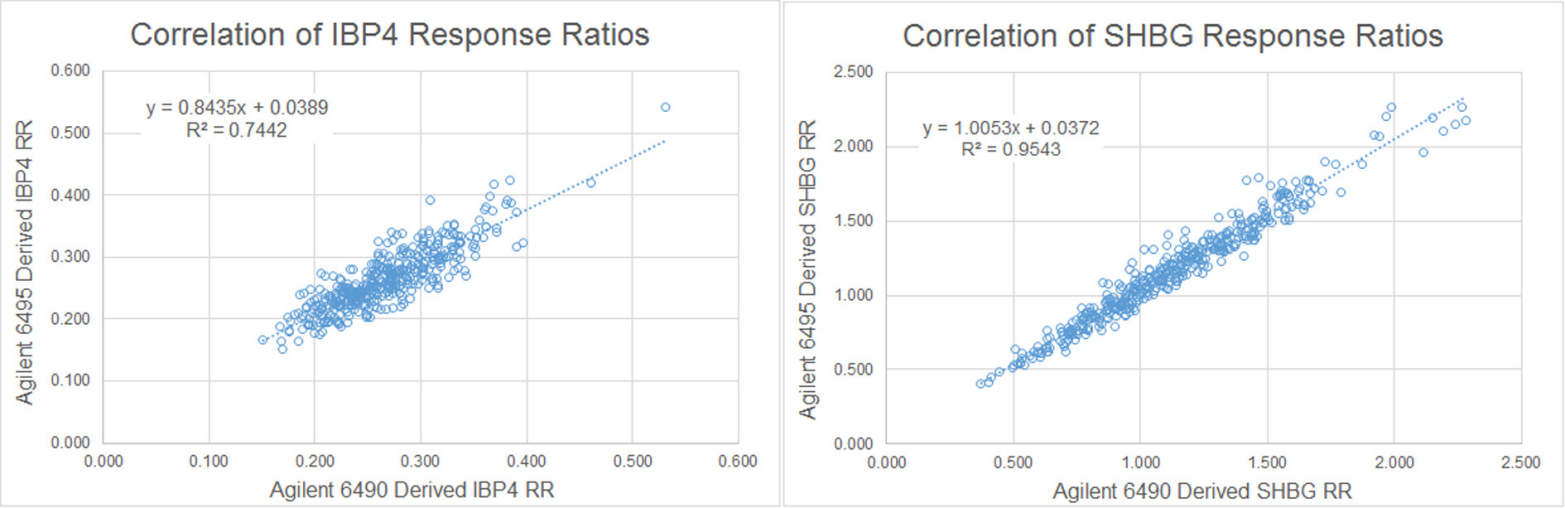
Alternative method comparison, response ratios.

**Fig. 5:**
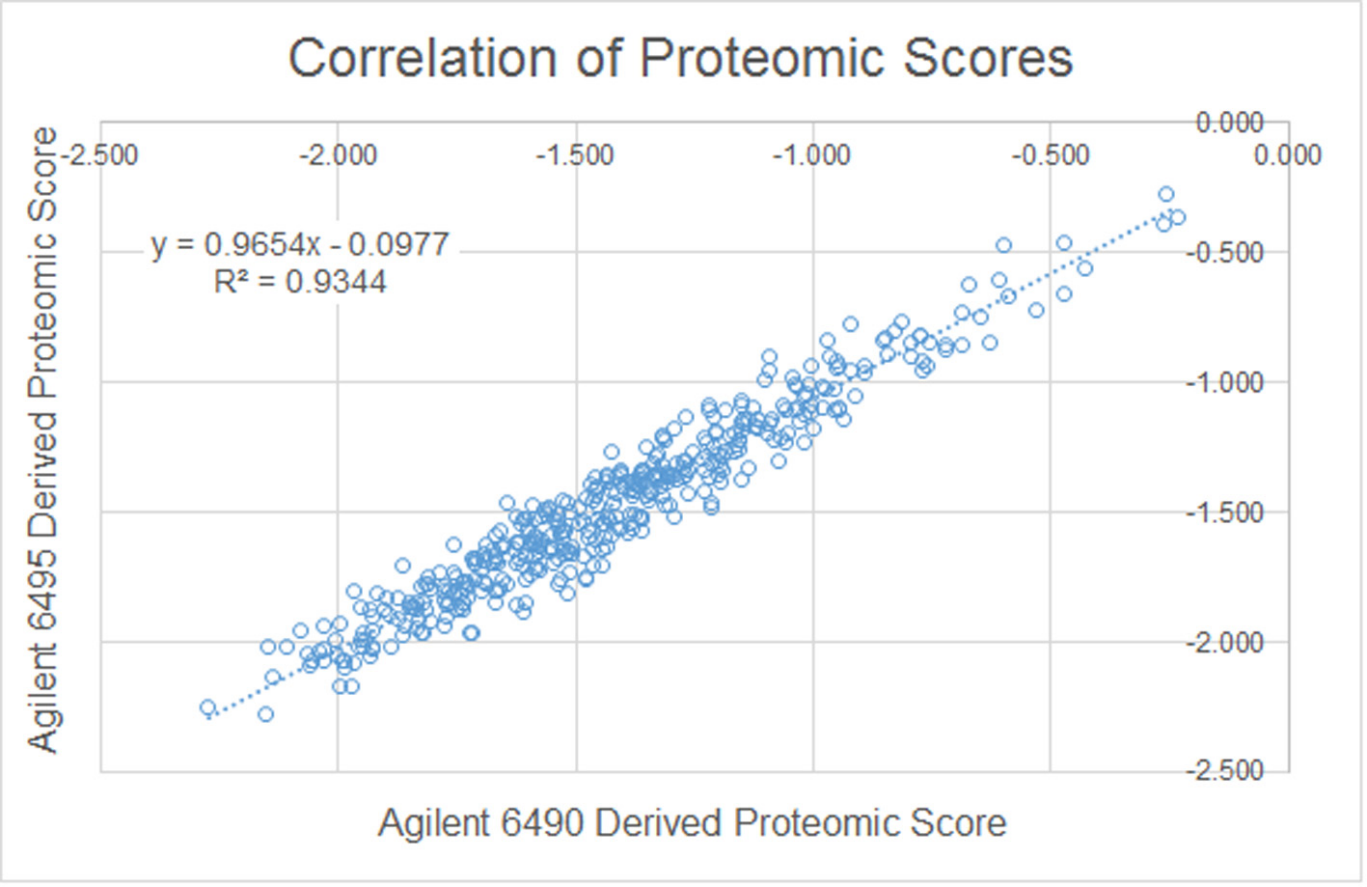
Alternative method comparison, proteomic score.

PAPR samples (n = 413) were used to verify that the second-generation alternative analytical method provided results that were accurate, relative to the first-generation reference method. Linear regression of each of the analyte response ratios, as well as the proteomic scores derived from the two models of LC-MS/MS systems, showed excellent correlation between the two methods (Fig. 4, Fig. 5) providing evidence that the alternative method maintains accuracy relative to the reference method and, consequently, yields equivalent test results. The assaying of the 413 samples occurred over a 43-day period.

### 3.3 Linearity

**Fig. 6:**
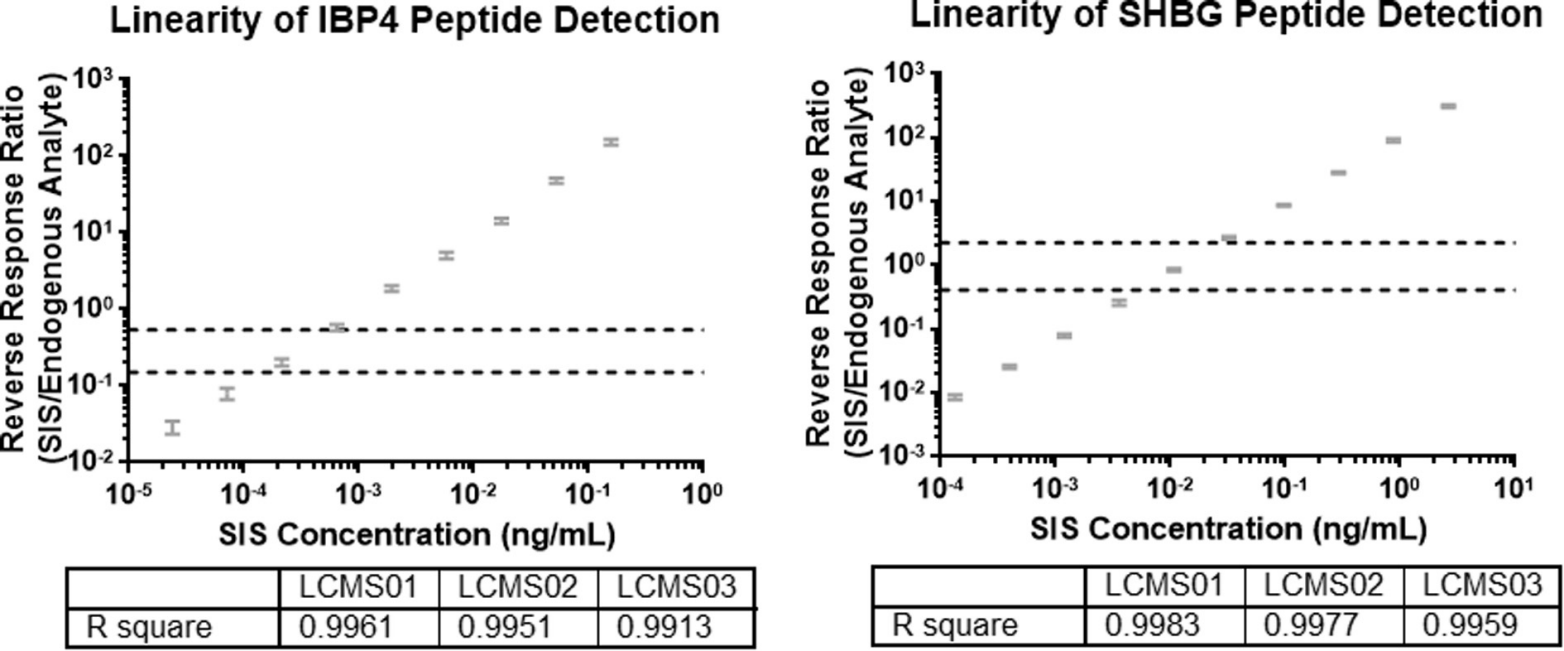
Peptide detection linearity.

Linearity samples were generated by maintaining the endogenous analyte response and fortifying the samples with increasing amounts of SIS. The SIS served as a surrogate for the detection of the endogenous analyte. The endogenous analyte response was kept constant and functioned by normalizing the SIS response. The response ratios used for linearity determination were generated by dividing the SIS response by the endogenous analyte response, the reverse of normal usage. This method, referred to as a reverse calibration curve [20], was used as a tool to determine if the detector generated a proportional response for the two signature analytes across a range of analyte abundances that are clinically relevant. The results from one of three detectors tested are shown in Fig. 6 (the results from the other two detectors are shown in the supplementary data section). Both signature analytes had a linear detector response across a broad range that encompassed the range of responses obtained from a large number of PAPR samples (n = 413), which represents the intended use population (dashed lines). The three LC-MS/MS instruments tested had R^2^ values that were >0.99 for IBP4 and SHBG.

### 3.4 Limits of quantitation

The lower and upper limits of quantitation were determined utilizing the same sample sets as used for the linearity study, and also the same reverse response ratio methodology. The limits of quantitation were established at the lower and upper extremes of the linear range in which the reversed response ratios maintained an acceptable level of precision of ≤20% [21]. The lower and upper limits of quantitation for the IBP4 peptide, as measured by a reverse response ratio, were 0.040 and 210, respectively (Table 6). The lower and upper limits of quantitation for the SHBG peptide, as measured by response ratio, were 0.011 and 291, respectively (Table 6). Carryover was below detection limits for IBP4 and was not significant for SHBG.

**Table 6:** Limits of quantitation.

Average IBP4 reverse response ratio	Reverse response ratio %CV			Average SHBG Reverse response ratio	Reverse response ratio %CV		
	LCMS01	LCMS02	LCMS03		LCMS01	LCMS02	LCMS03
0.019	13.21	*22.81*	*25.20*	0.011	4.18	3.51	7.88
0.040	10.64	8.06	18.99	0.031	3.58	3.82	6.05
0.099	7.67	10.61	17.58	0.093	3.90	4.17	5.67
0.261	5.94	7.75	11.10	0.290	2.40	3.57	9.23
0.768	4.85	4.59	9.15	0.889	3.44	4.40	5.05
2.349	4.90	6.56	8.72	2.769	4.40	3.54	5.34
6.910	4.39	6.61	10.39	8.660	3.37	1.86	3.70
20.161	3.65	7.70	8.10	27.147	4.57	3.56	3.89
64.870	3.88	5.29	7.78	85.917	2.29	2.93	5.24
209.911	5.21	6.22	8.52	291.267	1.86	2.71	4.43

Italicized values were over the 20% CV acceptance criteria.

### 3.5 Analytical specificity

The analytical specificity of the assay was attained from the high specificity intrinsic in LC-MS/MS-based detection. The retention time of each peptide was initially determined empirically on the analytical column. To confirm the measured signal was from the expected endogenous analyte, a heavy-labeled analogue with identical chemical properties to the endogenous peptide, but with a discernable signal because of the heavy labeling, was run simultaneously. At each peptide's determined retention time, the mass spectrometer was programmed to monitor two parent-product ion *m*/*z* transitions for each peptide and the supporting heavy-labeled analogue. The signal ratio of the two transitions (i.e., transition ratio) was calculated. Retention time, *m*/*z* of parent and product ions, and matching transition ratios between the endogenous peptide analyte and the exogenous heavy-labeled analogue were used to confirm each signal was, indeed, from the expected endogenous analyte.

**Fig. 7:**
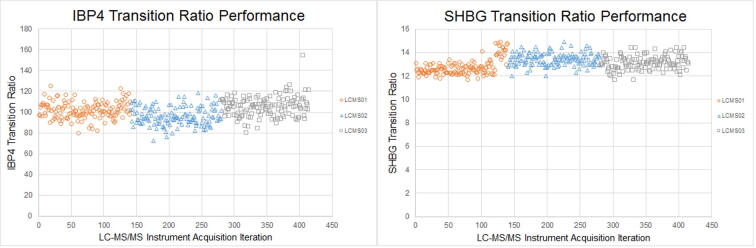
Transition ratio performance.

The signature peptide transition ratios measured in the 413 samples assayed over a 43-day period as part of the alternative method comparison did not have chromatographic anomalies (e.g., change in peak shape, significant increases in noise, split peaks), and only one sample had a transition ratio that was >±30% of the mean transition ratio (Fig. 7, [22]).

### 3.6 Endogenous interferent testing

At the concentrations tested, none of the endogenous interferents tested had a significant impact on the proteomic score calculated (Table 7). The concentrations of endogenous interferents tested were intentionally above the concentrations that would be expected in clinical samples to confirm robustness.

**Table 7:** Endogenous interferent results table.

Interferent	Tested Concentration	TTEST P-Value for Proteomic Score	TTEST P-Value for Risk Probability
Trigycleride Rich Lipoprotein	1372 mg/dL	0.12	0.16
Hemolysate	500 mg/dL	0.11	0.11
Protein	12 g/dL	0.47	0.34
Conjugated Bilirubin	20 mg/dL	0.58	0.75
Unconjugated Bilirubin	20 mg/dL	0.38	0.32

### 3.7 Analyte stability

The p-value of a paired *t*-test comparing the means of samples subjected to multiple freeze/thaw cycles with the means of the same samples that were not stressed was 0.23. The difference between a sample stressed with three freeze/thaw cycles was not significantly different than a non-stressed sample and, therefore, was considered stable for up to three freeze/thaw cycles. The stability of the signature analytes in stored samples was established through the consistent performance of the quality control materials stored for >2 years at −80 °C.

### 3.8 Quality control and long-term performance

The assay described in this study contains many steps towards the preparation of a sample that is assayed by LC-MS/MS. Each step could have been affected by environmental, procedural, reagent quality and stability, and equipment performance issues. Accordingly, several QC metrics were developed and adopted to monitor the performance of the assay for individual batches of samples, and across multiple batches, in order to assess long-term performance. Acceptability of any variances detected was judged according to the commonly applied Westgard rules [23].

**Fig. 8:**
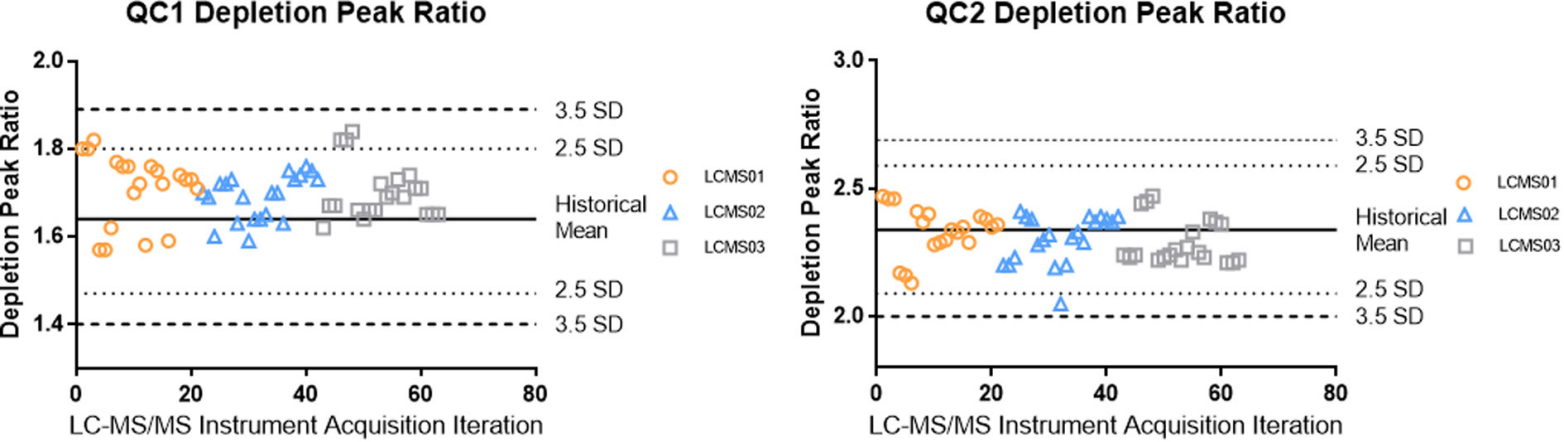
Depletion peak ratio performance.

The quality of a batch was determined using multiple replicates of two pooled serum quality control samples (i.e., QC1 and QC2) which bookended the clinical samples. The performance of QC1 and QC2 during depletion was monitored through the ratio of the chromatographic peak areas of the flow-through and depleted fractions in the 21 batches that were used for alternative method comparison, as shown in Fig. 8.

**Fig. 9:**
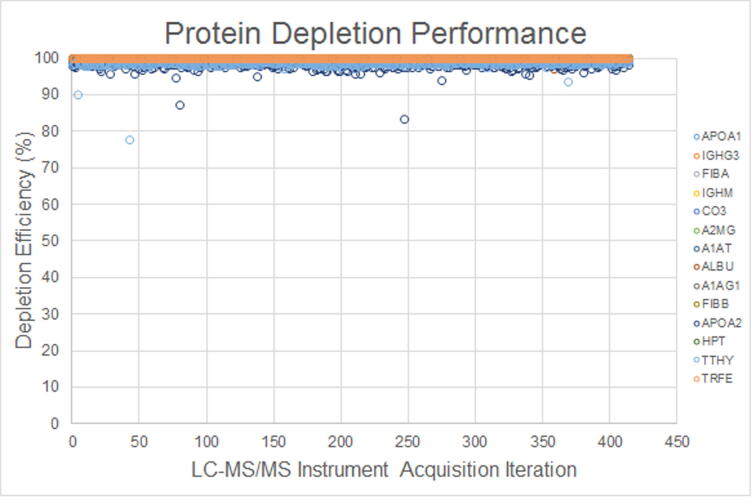
Depleted protein performance.

The protein depletion column depleted 98.0–99.9% of 14 proteins with high concentrations in human blood. Since the assay measured the amount of protein that was not depleted in a serum sample, a small change in depletion efficiency would have resulted in large changes in response ratios. For example, a sample that had 99.0% of apolipoprotein A1 (APOA1) depleted would have had a nearly ten-fold increase in response than a sample that had 99.9% depleted. A peptide from each of the 14 depleted proteins was assessed to support the efficiency of protein depletion. If a depleted protein exceeded a protein-specific threshold (i.e., <96–98% depletion efficiency) the sample and/or batch was subjected to a supervisory quality review. The responses for the depleted proteins measured in the 413 clinical samples used in the alternative method comparison are shown in Fig. 9. An occasional sample had reduced depletion efficiency of APOA1 and transthyretin (TTHY). During endogenous interferent testing, samples burdened with high concentrations of triglyceride-rich lipoprotein had APOA1 concentrations that were higher than the less efficiently depleted samples, without a significant impact on assay performance.

**Fig. 10:**
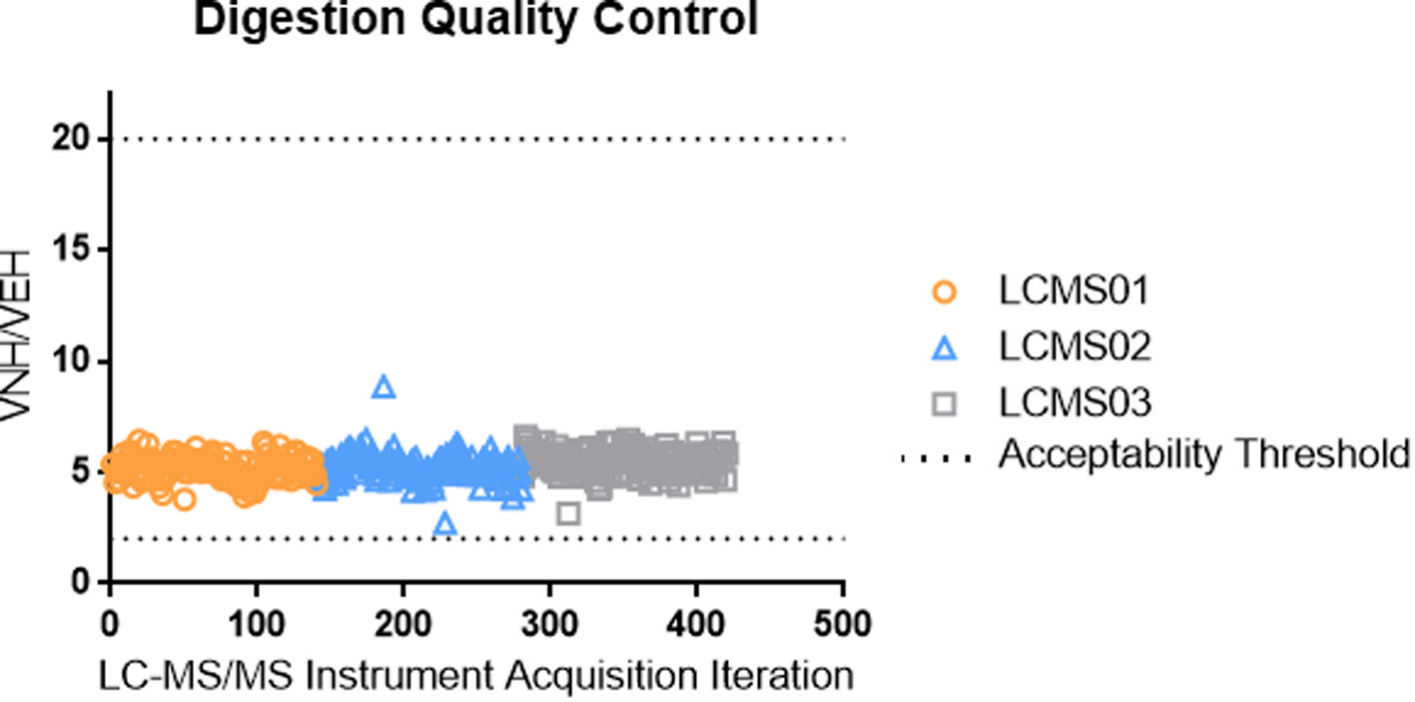
Digestion quality control performance.

Two peptides for beta 2-microglobulin with different trypsin kinetic profiles were monitored to support the quality of trypsin digestion. If the kinetics of trypsin digestion were changed for a sample or a batch, then the ratio of the chromatographic peak areas for these two peptides would fall outside of the acceptance range and the individual sample, or the batch, would be failed. The digestion quality control results for the 413 samples assayed as part of alternative method comparison are shown in Fig. 10. In this figure, the x-axis represents the alternative method comparison samples arranged by analysis date, and the y-axis represents the ratio obtained from the chromatographic peak areas of the two beta 2-microglobulin peptides (amino acid sequence of VNHVTLSQPK and VEHSDLSFSK). The lower and upper acceptance thresholds are marked with a dashed line.

**Fig. 11:**
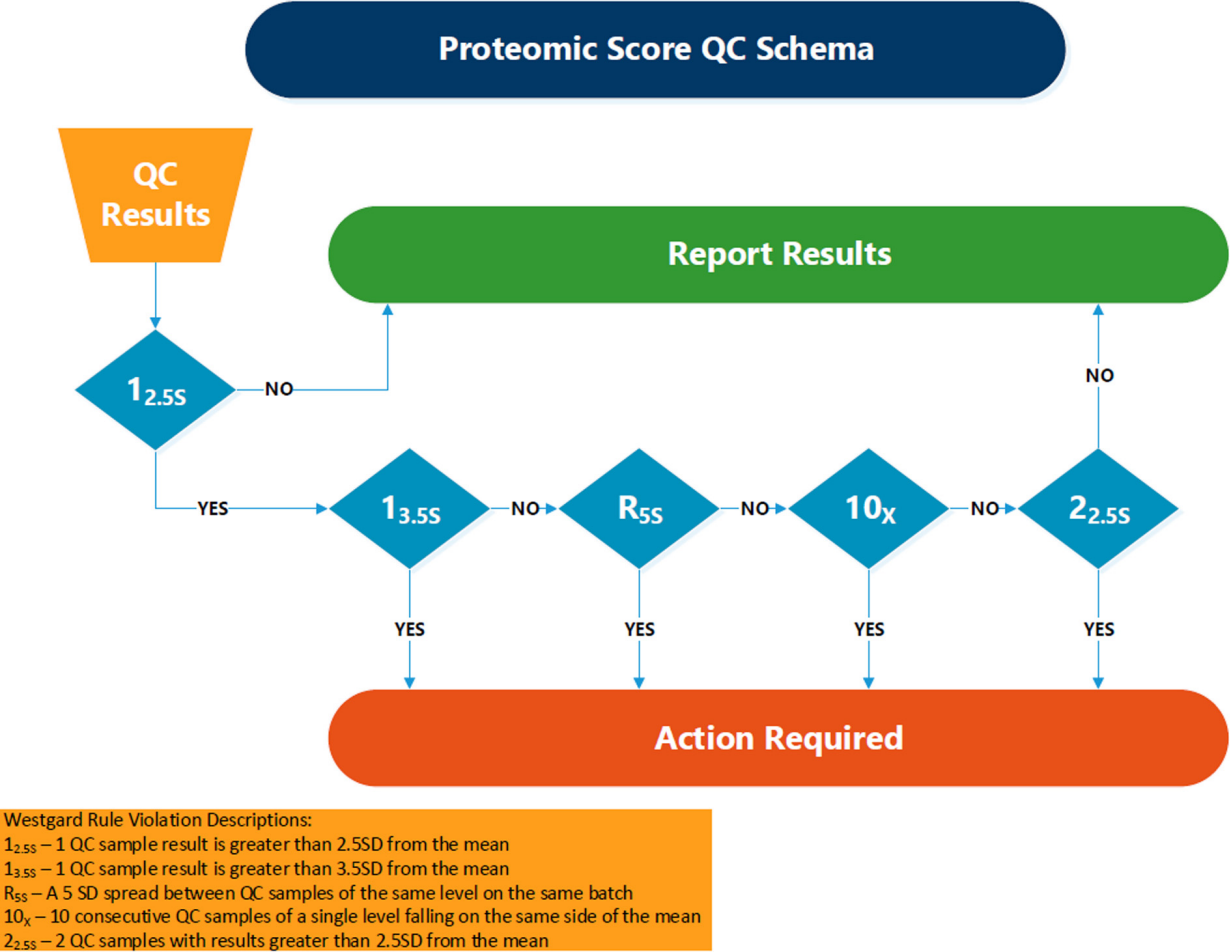
Proteomic score-based batch quality control flowchart.

The quality of the assay was also determined through interrogation of the quality control samples. The proteomic score for each individual quality control sample and the batch mean for a quality control sample type were acceptable if they were ≤2.5 standard deviations from the historical mean. If a quality control was ≥2.5 standard deviations from the historical mean, then a cascade of Westgard rules [23] was applied: an additional quality control sample that was ≥2.5 standard deviations from the historical mean resulted in batch failure; a maximum range of proteomic scores for a quality control sample type that was ≥5 standard deviations from the historical mean resulted in batch failure; ten consecutive replicates of a quality control sample type on one side of the historical mean resulted in batch failure. Any batch that had a quality control sample that was ≥3.5 standard deviations of the historical mean was failed. A flowchart of the proteomic score-based batch quality control is shown in Fig. 11.

**Fig. 12:**
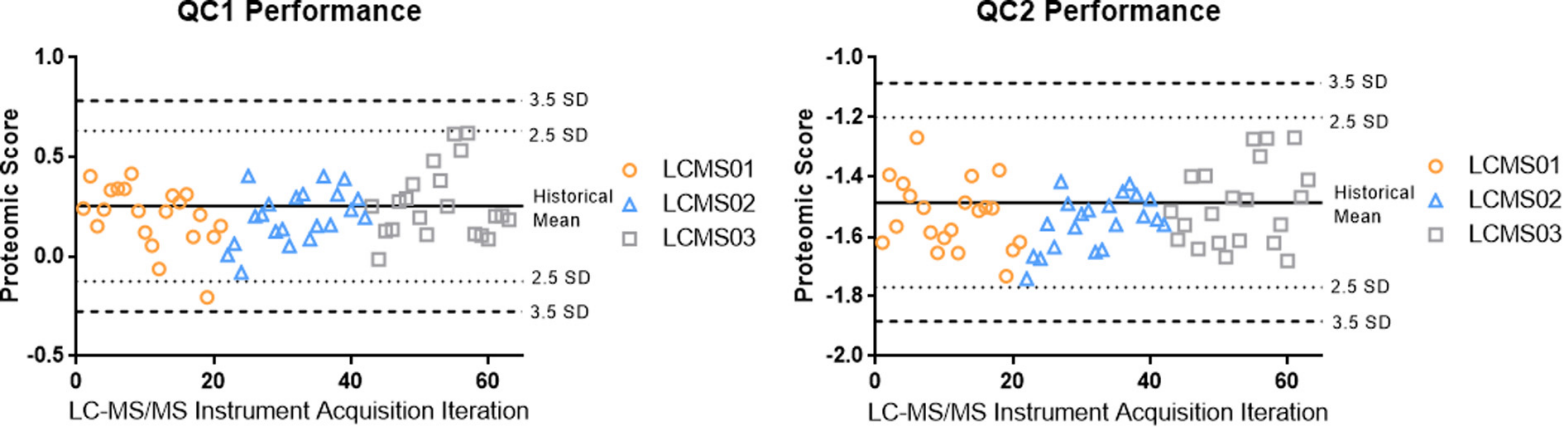
Proteomic score performance.

The performance of the QC1 and QC2 during the analysis of 413 clinical samples that comprised alternative method comparison are shown in Fig. 12. The proteomic scores for the two quality control samples were within 2.5 SD of the historical mean, except for one QC1 replicate, providing support for the quality of the clinical samples run within those batches.

## 4 Conclusions

A targeted proteomic workflow coupled with LC-MS/MS-based detection of peptides for two protein biomarkers of sPTB was validated. The validated method has inter- and intra-batch precision that generates agreeable results over time and across multiple sets of instrumentation. The validated method is a novel second-generation method that produces accurate results relative to the reference first-generation method. The detector response for each of the signature peptides is linear across several orders of magnitude and encompasses the range of responses expected for the intended population. Limits of quantitation were determined, defining a range of analyte responses with acceptable performance. The level of analyte carryover in the method is insignificant. The method is resilient to the differences in sample matrix, high levels of endogenous interferents, and stress incurred during repeated freeze/thaw cycles and long-term storage. Lastly, quality metrics developed to monitor the assay enabled the assessment of individual batch process quality and long-term trend analyses. Together, these results demonstrate the acceptable performance and robustness of an analytical method that provides relative abundances of maternal serum proteins needed to generate a qualitative individualized determination of a woman’s risk of sPTB. As such, the two analytical methods yield equivalent test results.

Additional opportunities exist for improvement of assay efficiency. These include a further reduction of sample complexity through affinity enrichment techniques, at either the protein or peptide level. Such simplification would potentially allow for even shorter LC-MS/MS run times with a commensurate increase in throughput.

The authors would like to acknowledge the contributions made during the initial phase of this work by Pascal Croteau, Jeff Flick, Laura McIntosh, Warren Porter, and Michael Schrim.

## Funding source

Sera Prognostics, Inc. provided the funding necessary for the research, method development, assay validation, data analysis and interpretation, and drafting of this submission.

## Conflict of interest

All authors associated with Sera Prognostics, Inc. are or have been employees of Sera Prognostics, Inc. and as such receive monetary compensation. Additionally, all authors associated with Sera Prognostics, Inc. receive or have received options to purchase shares of the company at a preferred price. All authors associated with Integrated Diagnostics, Inc. are or have been paid consultants to Sera Prognostics, Inc. All authors associated with Caprion are or have been employed with Caprion, a company which Sera Prognostics, Inc. has utilized as a service provider.

## Figures and Tables

**Table:** 

Protein	Proteotypic Peptide	Analyte Type	Transition	Retention Time (min)	Collision Energy	Dwell Time (ms)	Peptide Transition	SIS Transition
IBP4	QCHPALDGQR	Signature	Quantitative	2.64	11	42	394.5_475.2	397.9_485.2
IBP4	QCHPALDGQR	Signature	Qualitative	2.64	17	42	394.5_360.2	397.9_370.2
SHBG	IALGGLLFPASNLR	Signature	Quantitative	10.82	13	50	481.3_657.4	484.6_667.4
SHBG	IALGGLLFPASNLR	Signature	Qualitative	10.82	17	50	481.3_412.3	484.6_412.3
APOA1	AKPALEDLR	Depleted	Quantitative	5.22	13	11	506.8_813.5	511.8_823.5
APOA1	AKPALEDLR	Depleted	Qualitative	5.23	21	11	506.8_288.2	511.8_298.2
IGHG3	ALPAPIEK	Depleted	Quantitative	5.77	8	12	419.8_327.7	423.8_331.7
IGHG3	ALPAPIEK	Depleted	Qualitative	5.77	8	12	419.8_654.4	423.8_662.4
FIBA	ESSSHHPGIAEFPSR	Depleted	Quantitative	5.26	7	11	546.6_502.2	549.9_502.2
FIBA	ESSSHHPGIAEFPSR	Depleted	Qualitative	5.26	11	11	546.6_353.7	549.9_358.7
IGHM	GFPSVLR	Depleted	Quantitative	7.19	10	8	388.2_286.2	393.2_291.2
IGHM	GFPSVLR	Depleted	Qualitative	7.19	10	8	388.2_571.4	393.2_581.4
CO3	IHWESASLLR	Depleted	Quantitative	7.43	21	12	606.3_251.2	611.3_251.2
CO3	IHWESASLLR	Depleted	Qualitative	7.43	21	12	606.3_437.2	611.3_437.2
A2MG	LHTEAQIQEEGTVVELTGR	Depleted	Quantitative	7.34	25	10	704.0_680.3	707.4_684.4
A2MG	LHTEAQIQEEGTVVELTGR	Depleted	Qualitative	7.34	17	10	704.0_674.4	707.4_680.3
A1AT	LSITGTYDLK	Depleted	Quantitative	7.60	15	11	555.8_797.4	559.8_805.4
A1AT	LSITGTYDLK	Depleted	Qualitative	7.60	15	11	555.8_696.4	559.8_704.4
ALBU	LVTDLTK	Depleted	Quantitative	5.30	10	11	395.2_577.3	399.2_585.3
ALBU	LVTDLTK	Depleted	Qualitative	5.30	7	11	395.2_213.2	399.2_213.2
A1AG	NWGLSVYADKPETTK	Depleted	Quantitative	7.15	20	8	570.3_301.1	573.0_301.1
A1AG	NWGLSVYADKPETTK	Depleted	Qualitative	7.15	12	8	570.3_818.4	573.0_826.4
FIBB	QGFGNVATNTDGK	Depleted	Quantitative	4.81	19	13	654.8_706.3	658.8_714.4
FIBB	QGFGNVATNTDGK	Depleted	Qualitative	4.81	27	13	654.8_319.2	658.8_327.2
APOA2	SPELQAEAK	Depleted	Quantitative	3.55	13	31	486.8_788.4	490.8_796.4
APOA2	SPELQAEAK	Depleted	Qualitative	3.55	13	31	486.8_659.4	490.8_667.4
HPT	TEGDGVYTLNNEK	Depleted	Quantitative	5.32	21	12	720.3_881.4	724.3_889.5
HPT	TEGDGVYTLNNEK	Depleted	Qualitative	5.32	21	12	720.3_403.2	724.3_403.2
TTHY	VEIDTK	Depleted	Quantitative	3.05	8	29	352.7_476.3	356.7_484.3
TTHY	VEIDTK	Depleted	Qualitative	3.05	8	29	352.7_605.3	356.7_613.3
TRFE	YLGEEYVK	Depleted	Quantitative	6.62	13	17	500.8_724.4	504.8_732.4
TRFE	YLGEEYVK	Depleted	Qualitative	6.62	9	17	500.8_277.2	504.8_277.2
B2M	VEHSDLSFSK	Digestion QC	Quantitative	4.78	9	16	383.5_468.2	386.2_476.3
B2M	VEHSDLSFSK	Digestion QC	Qualitative	4.78	9	16	385.5_234.1	386.2_242.2
B2M	VNHVTLSQPK	Digestion QC	Quantitative	4.00	9	30	374.9_244.2	377.6_252.2
B2M	VNHVTLSQPK	Digestion QC	Qualitative	4.00	14	30	374.9_459.3	377.6_467.3

## Glossary

Alternative Method Comparison: A performance comparison between a reference method and a new candidate alternative method using a set of samples
Carryover: Materials from a sample that remain in a system and are introduced into subsequent samples
IBP4: Insulin-like growth factor-binding protein 4
LC-MS/MS: An analytical chemistry technique that combines the physical separation capabilities of liquid chromatography (LC) with the mass analysis capabilities of tandem mass spectrometry (MS/MS)
Limit of Quantitation: A lower or upper limit of analyte abundance where expected assay performance is maintained
Linearity: The ability of an assay to generate results that are proportional to a given range of relative abundances of the analyte(s) being measured
Precision: The extent an assay can generate agreeable results on a sample set over time using multiple instrumentation clusters, multiple lots of reagents, and multiple operators
Protein Depletion: The specific depletion of high abundance proteins in serum using an immuno-affinity material
Proteotypic Peptide: A peptide that is unique to a protein.
PTB: Preterm birth, any delivery that is <37 weeks gestation
Qualitative Transition: The transition used to support the trueness of measurement of the quantitative peak, typically the transition that provides the lower response
Quantitative Transition: The primary transition used for measuring an analyte, typically the transition that provides the higher response
Response Ratio: The ratio generated by dividing the area counts for the quantitative analyte transition by the quantitative SIS transition
SHBG: Sex hormone-binding globulin
SIS: Stable isotope standard, used for normalizing the response of the light endogenous analyte
sPTB: Spontaneous preterm birth, a preterm birth that is not iatrogenic (or “indicated”)
Transition Ratio: The ratio of the qualitative transition area count divided by the quantitative transition area count, multiplied by 100
