# Supplementary figures and images for: Analytical validation of protein biomarkers for risk of spontaneous preterm birth
Source: Clin Mass Spectrom. 2017 Jun 12;3:25–38. doi: 10.1016/j.clinms.2017.06.002 (PMC11322780; doi:10.1016/j.clinms.2017.06.002)

# Supplementary Material

Figure 13 - Peptide Detection Linearity


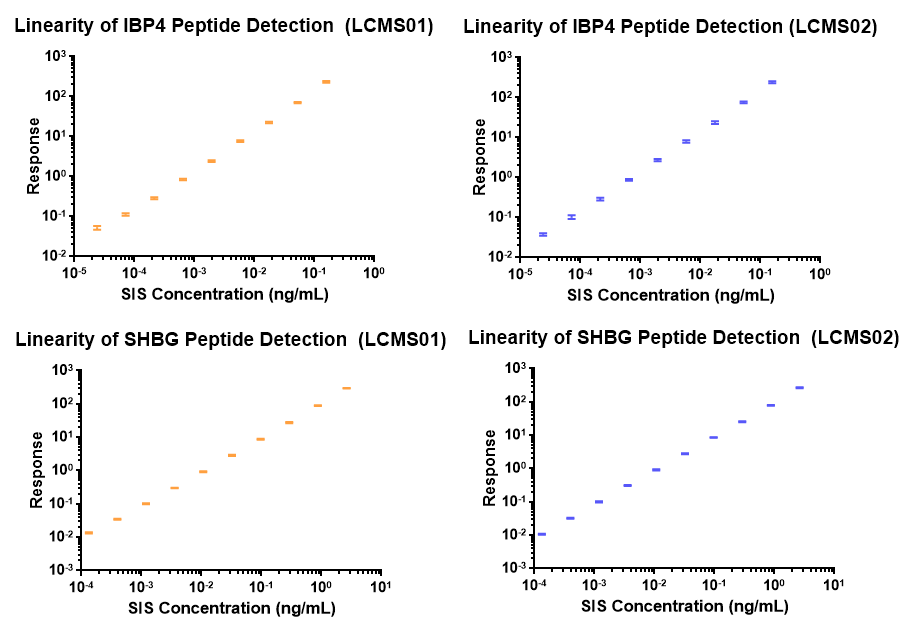

Supplement: Supplementary data [file mmc1.docx]
